# Dual Testing Algorithm of BED-CEIA and AxSYM Avidity Index Assays Performs Best in Identifying Recent HIV Infection in a Sample of Rwandan Sex Workers

**DOI:** 10.1371/journal.pone.0018402

**Published:** 2011-04-12

**Authors:** Sarah L. Braunstein, Denis Nash, Andrea A. Kim, Ken Ford, Lambert Mwambarangwe, Chantal M. Ingabire, Joseph Vyankandondera, Janneke H. H. M. van de Wijgert

**Affiliations:** 1 Mailman School of Public Health-Columbia University, New York, New York, United States of America; 2 Centers for Disease Control and Prevention, Atlanta, Georgia, United States of America; 3 Projet Ubuzima, Kigali, Rwanda; 4 Belgian Development Agency, Kigali, Rwanda; 5 Department of Internal Medicine, and Amsterdam Institute for Global Health and Development, Academic Medical Center of the University of Amsterdam, Amsterdam, The Netherlands; McGill University, Canada

## Abstract

**Background:**

To assess the performance of BED-CEIA (BED) and AxSYM Avidity Index (Ax-AI) assays in estimating HIV incidence among female sex workers (FSW) in Kigali, Rwanda.

**Methodology and Findings:**

Eight hundred FSW of unknown HIV status were HIV tested; HIV-positive women had BED and Ax-AI testing at baseline and ≥12 months later to estimate assay false-recent rates (FRR). STARHS-based HIV incidence was estimated using the McWalter/Welte formula, and adjusted with locally derived FRR and CD4 results. HIV incidence and local assay window periods were estimated from a prospective cohort of FSW. At baseline, 190 HIV-positive women were BED and Ax-AI tested; 23 were classified as recent infection (RI). Assay FRR with 95% confidence intervals were: 3.6% (1.2–8.1) (BED); 10.6% (6.1–17.0) (Ax-AI); and 2.1% (0.4–6.1) (BED/Ax-AI combined). After FRR-adjustment, incidence estimates by BED, Ax-AI, and BED/Ax-AI were: 5.5/100 person-years (95% CI 2.2–8.7); 7.7 (3.2–12.3); and 4.4 (1.4–7.3). After CD4-adjustment, BED, Ax-AI, and BED/Ax-AI incidence estimates were: 5.6 (2.6–8.6); 9.7 (5.0–14.4); and 4.7 (2.0–7.5). HIV incidence rates in the first and second 6 months of the cohort were 4.6 (1.6–7.7) and 2.2 (0.1–4.4).

**Conclusions:**

Adjusted incidence estimates by BED/Ax-AI combined were similar to incidence in the first 6 months of the cohort. Furthermore, false-recent rate on the combined BED/Ax-AI algorithm was low and substantially lower than for either assay alone. Improved assay specificity with time since seroconversion suggests that specificity would be higher in population-based testing where more individuals have long-term infection.

## Introduction

The Serologic Testing Algorithm for Recent HIV Seroconversion (STARHS) offers a promising alternative to prospective measurement of HIV incidence, particularly in developing countries where incidence rates may be high but are infrequently measured owing to limited resources[1]–[4]. Two main STARHS assays are the BED capture enzyme immunoassay (BED)[5] and AxSYM Avidity Index method (Ax–AI)[6]. These and other STARHS assays exploit biologic properties of early HIV infection, such as development of HIV antibodies, to distinguish recent from long-term infections in cross-sectional samples of individuals testing HIV positive.

Studies conducted in a range of populations and settings, however, reveal the tendency of STARHS assays, including the BED and Ax-AI, to misclassify certain individuals with long-term HIV infection as recently infected, thus inflating HIV incidence estimates relative to prospective cohort rates[5], [7]–[11]. A number of strategies have been proposed for correcting assay misclassification, including statistical adjustment, assessment of CD4 count and antiretroviral therapy (ART) status among individuals tested in order to remove those with probable long-term infection (LTI) from “recent infection” (RI) classification by the assays prior to calculation of incidence, and use of a dual testing algorithm in which a second, different STARHS assay confirms the classification on an initial assay[8], [9], [12]–[17]. In addition, individuals who test HIV-positive in a cross-sectional survey can be followed in a “long-term infection cohort” with repeat STARHS testing ≥12 months later, in order to calculate assay false-recent rates (FRR); incidence estimates can then be adjusted downward by applying the FRR to available statistical formulae[8], [9], [18].

We applied the BED and Ax-AI assays in a cross-sectional survey and to post-seroconversion panel specimens from a prospective HIV seroconversion study among female sex workers (FSW) at Projet Ubuzima in Kigali, Rwanda. This paper reports on the proportion of samples testing RI; concordance between BED and Ax-AI results; frequency and factors associated with false-recent classifications by the assays; estimated mean window periods for the assays based on data from cohort seroconverters; estimated HIV incidence in the prospective cohort; and unadjusted and adjusted STARHS-based incidence estimates for the cross-sectional sample.

## Materials and Methods

Eight hundred FSW 18 years and older were HIV tested in a cross-sectional survey. All women provided written informed consent prior to study participation. Women who tested HIV positive during the survey were further tested by the BED and Ax-AI assays, and then asked to return to the study clinic at least 12 months later for repeat testing by both assays. HIV-negative, non-pregnant women were eligible to enroll in a prospective HIV seroconversion cohort. Cohort participants (N = 397) returned for quarterly follow-up visits for one year and then for a single visit during the second year of follow-up.

HIV testing was by First Response Rapid Test (Premier Medical Corporation, India) and Uni-Gold Rapid Test (Trinity Biotech Plc, Ireland), with Capillus HIV-1/HIV-2 Rapid Test (Trinity Biotech Plc, Ireland) as the tie-breaker. Rapid test-positive results were confirmed by Murex HIV Ag/Ab Combination ELISA (Abbott Laboratories, Germany), and tested by CD4 cytometry. Rapid test-negative specimens were pooled for testing by HIV-1 RNA PCR to identify acute HIV infections (COBAS TaqMan, Roche Molecular Systems, Inc., USA).

Specimens from participants testing HIV positive in the cross-sectional survey and cohort were tested with the BED and Ax-AI assays. The BED assay measures the ratio of HIV-specific immunoglobulin (IgG) antibody to total antibody; a low proportion indicates infection within the past 155 days (i.e., RI) (95% confidence interval [CI]: 146–165)) [2], [5]. BED testing was performed onsite following the manufacturer's package insert (Calypte® Biomedical Corporation, Oregon, US)[19]. The Ax-AI method measures the “avidity”—or strength—of the HIV antibody-antigen bond; avidity is weak among individuals infected during the past 180 days (i.e., RI)[6], [20]. Avidity testing was performed by the Pediatric HIV Research Unit in South Africa using the AxSYM HIV-1/2gO ELISA (Abbott, USA), and following procedures described elsewhere[6], [20].

Women who tested HIV positive were given CD4 results and referred for HIV care and evaluation for treatment eligibility, as well as psychosocial services. BED and Ax-AI results were not given to participants, as the assays are designed for research purposes only[21]. The National Ethics Committee and the National AIDS Control Commission (CNLS) in Rwanda, and the Columbia University Medical Center Institutional Review Board in the United States, approved the study.

### Statistical methods

#### Prospective cohort sample

The estimated cohort HIV incidence rate and 95% CI were calculated using standard incidence formulae, assuming a Poisson distribution. HIV infection was assumed to have occurred at the midpoint between the last negative HIV test and first positive HIV test.

We estimated study sample-specific mean window periods for the BED and Ax-AI assays, and combined BED/Ax-AI algorithm. While rigorous statistical methods, such as mixed effects regression or survival analysis techniques, are the preferred method for deriving mean assay window periods (see e.g., [22]), such methods require substantially larger sample sizes than were available for this analysis. Instead, we used an approximate method to estimate assay window periods: we observed in the pattern of antibody kinetics the point at which each subject crossed the assay cutoff (0.8 for BED, 0.85 for Ax-AI (*Ax-AI cutoff based on personal communication with B. Suligoi*)), and then averaged the values across subjects to obtain the mean assay window period. To estimate the window period for the combined BED/Ax-AI algorithm, we took the value of the earlier of the two assay threshold crossings (BED or Ax-AI) for each individual, and then averaged across individuals. For all window period calculations, we excluded seroconverters who self-reported initiating ART after HIV diagnosis, individuals lacking additional serial assay results from post-seroconversion visits, and those who did not reach the threshold of either assay during follow-up testing. Standard errors (SE) were calculated around window period estimates using standard spreadsheet software (Microsoft Excel, 2003).

#### Cross-sectional survey sample

In the cross-sectional sample, we calculated the proportion of HIV-infected participants classified as RI and LTI by each assay, as well as the proportion with concordant and discordant results on the two assays. A Kappa coefficient with 95% CI was calculated to measure agreement between BED and Ax-AI classifications.

HIV-1 incidence estimates and 95% CI based on the BED and Ax-AI assay results (with BED OD-n≤0.80 and Ax-AI≤0.85 indicating recent infection) were calculated using the formula, and accompanying spreadsheet (available at: http://www.sacema.com/page/assay-based-incidence-estimation; accessed July 8, 2010), provided by McWalter and Welter[23]. Inputs in the formula include the number of positive individuals in the sample, the number of recent infections, the assay window period (sample-specific estimates), and the number of HIV-negative individuals tested. Incidence estimates are expressed as an incidence rate (number of new HIV infections per 100 person-years), and confidence intervals are calculated using a delta method approximation. Incidence estimates were adjusted with CD4 count data, by excluding individuals with probable long-term infection (based on CD4<200) from recent infection classifications for incidence estimate calculations (these individuals were also excluded from FRR calculations). Further, a separate set of adjusted incidence estimates was generated by adjusting estimates with study-specific FRR for the BED and Ax-AI assays, and combined BED/Ax-AI algorithm. False-recent rate was defined as the proportion of ART-naïve, HIV-infected cross-sectional survey participants with known long-term infection and CD4 count ≥200 cells/µl who were classified by the BED and/or Ax-AI assay as having recent infection upon repeat testing ≥12 months later[8], [9].

All statistical analyses were performed using SAS version 9.2 (SAS Institute, Inc., Cary, NC).

## Results

### Assay results among prospective seroconverters

Nineteen individuals HIV seroconverted during the prospective cohort study, generating a total of 52 samples (19 from the seroconversion visit, and 33 post-seroconversion samples). The duration between seroconverters' last negative and first positive HIV test ranged from 83 to 406 days (mean: 204 days; median: 93 days). Among 16 of 19 seroconverters with CD4 data available within 3 months of the seroconversion visit, median CD4 count was 549 cells/µl (range: 287–1218).

**Figure 1:**
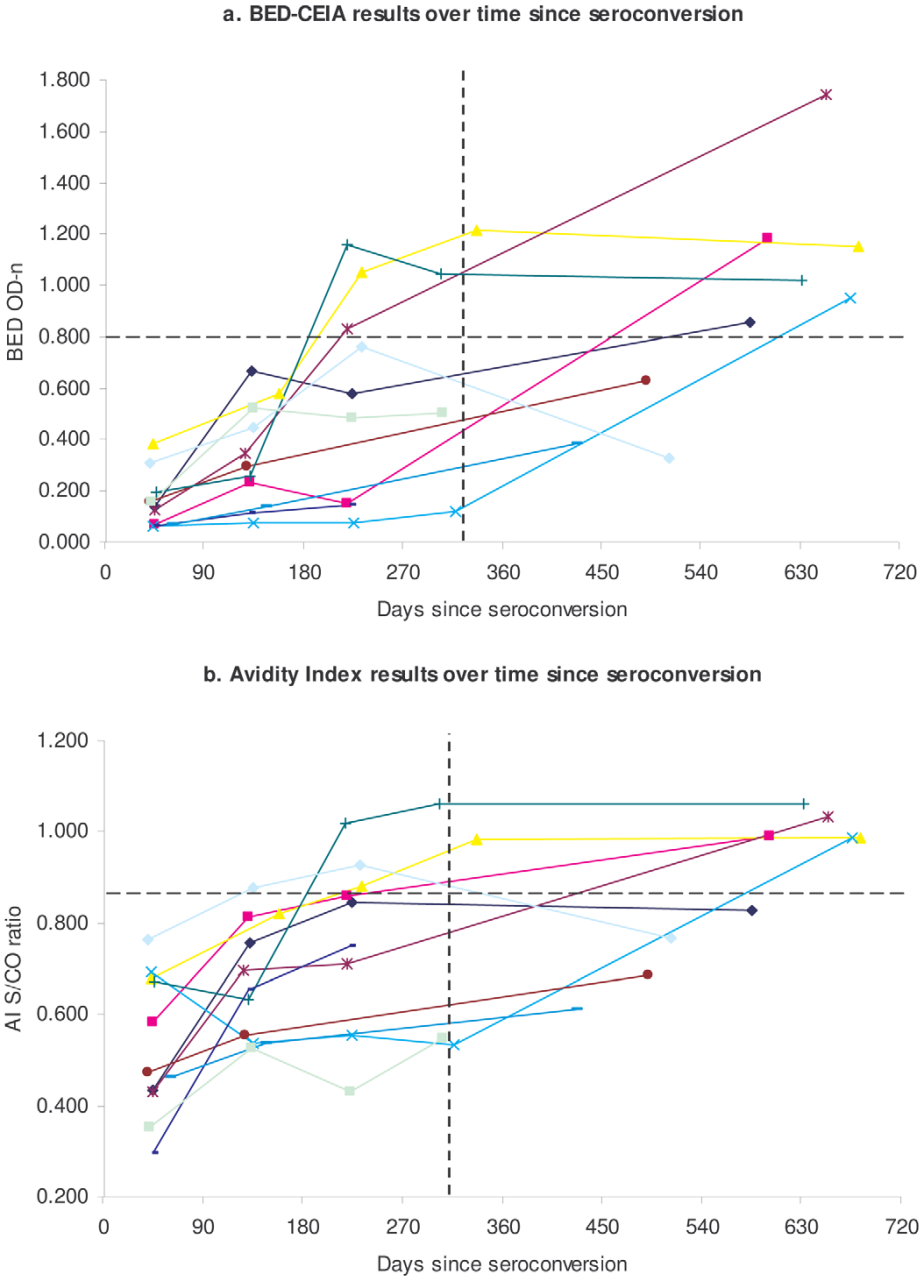
STARHS assay results over time among participants with incident HIV in prospective cohort (N = 11).

Figures 1a–b display BED OD-n values and Ax-AI scores, respectively, over time since HIV seroconversion among prospective cohort seroconverters (N = 11) with BED/Ax-AI results for post-seroconversion study visits. Among these 11 participants, 6 saw their BED result cross the designated assay cutoff value during follow-up, and 7 saw their Ax-AI result cross the cutoff. Using data from these participants whose infection status changed from RI to LTI during follow-up, and excluding data from 2 seroconverters who reported initiating ART since their HIV diagnosis, the estimated mean window periods for BED and Ax-AI were approximately 330 (SE 84.1) and 310 (SE 69.9) days, respectively. The estimated window period for the combined BED/Ax-AI algorithm was 267 days (SE 64.8).

### Assay classifications in the cross-sectional sample

**Figure 2:**
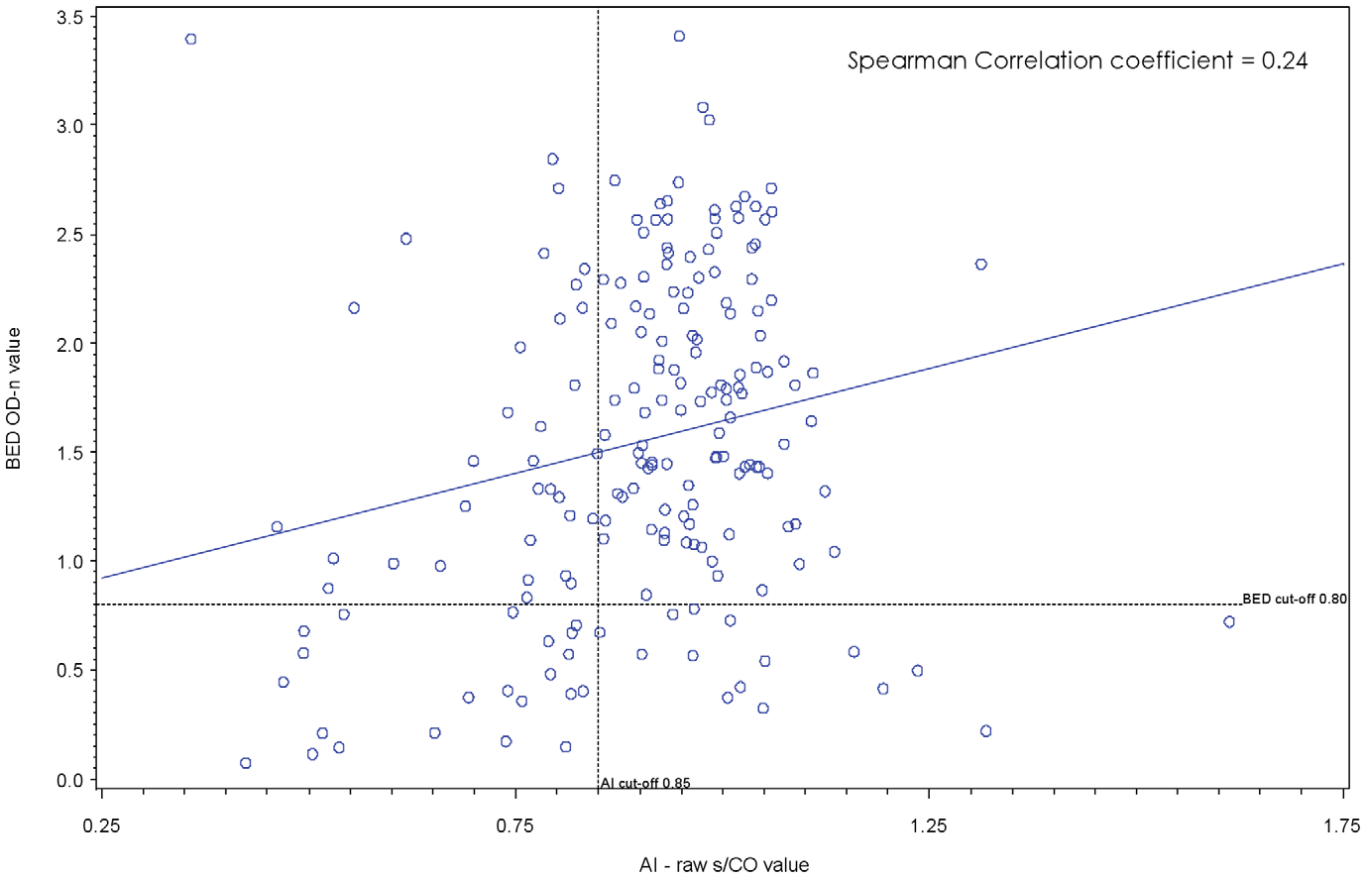
Correlation of results on BED-CEIA and AxSYM Avidity Index for recent HIV infection, Cross-sectional sample (N = 190).

A total of 192 women tested HIV positive in the cross-sectional survey (no acute HIV infections were identified by PCR). As none of the women knew their positive HIV serostatus prior to testing, all participants were ART-naïve at the time of the survey. Among HIV-positive participants, 190 were tested by BED and Ax-AI (Fig. 2): 36 (19%) were classified as RI by BED, and 56 (30%) as RI by Ax-AI; 23 (12%) were classified as RI by both assays; 121 (64%) were classified as LTI by both assays; and 46 (24%) were classified discordantly by the assays. BED and Ax-AI classifications were concordant for 76% of cross-sectional sample specimens, yielding a Kappa score of 0.35 (95% CI: 0.20, 0.50). The Spearman coefficient for correlation between BED OD-n and Ax-AI results was 0.24.

### False-recent rates

Among the 190 participants with STARHS results from the survey, 141 (74%) returned for repeat testing by BED and Ax-AI ≥12 months later (median 623 days, range: 432–861). Upon repeat testing, 80% (113/141) of women were correctly classified as having LTI by both assays. However, 9 women were falsely classified by BED as RI; 23 women were falsely classified by Ax-AI as RI; and 4 women were falsely classified by both assays as RI. Four of the 9 participants with BED-false recent results (44%), 8 of the 23 participants with Ax-AI-false recent results (35%), and 1 of the 4 participants with false recent results on the BED/Ax-AI algorithm (33%), reported taking ART since their HIV diagnosis. After excluding ART-positive individuals, FRR for the BED and Ax-AI were 3.6% (95% CI: 1.2–8.1) and 10.6% (95% CI: 6.1–17.0), respectively. The FRR for the combined BED/Ax-AI algorithm was 2.1% (95% CI: 0.4–6.1).

### Characteristics of individuals testing false-recent on assays

There was no significant association between testing false-recent on STARHS ≥12 months after HIV diagnosis and marital status, duration between baseline and repeat STARHS tests, years working as a sex worker, or recent AIDS-like symptom (Table 1). However, there was a borderline significant association between older age and higher likelihood of testing false-recent on the Ax-AI assay (*P* = 0.06). Furthermore, participants with a false-recent result on the Ax-AI assay had a significantly higher median baseline CD4 count than those correctly classified by Ax-AI (590 vs. 444 cells/µl, *P* <0.01). Median baseline CD4 cell count did not differ between participants falsely and correctly classified by the BED assay (447 vs. 461 cells/µl, *P* = 0.77). Moreover, on both assays, participants with a false-recent test result had been HIV tested more frequently in their lifetimes compared with participants who were correctly classified as LTI by the assays (BED *P* = 0.01, Ax-AI *P* = 0.02). Finally, on both assays, testing false-recent was significantly associated with having been classified as RI by the assay during the cross-sectional survey (BED *P*<0.0001, Ax-AI *P*<0.0001).

**Table 1:** Characteristics of cross-sectional survey participants with long-term HIV infection by repeat STARHS test result ≥12 months after HIV diagnosis (N = 141).

Characteristic	BED-CEIA Assay			Avidity Index method		
	False-recent result (n = 9)	Correctly classified as LTI (n = 132)	*P value* 1	False-recent result (n = 23)	Correctly classified as LTI (n = 118)	*P value* 1
Median age in years (IQR)	26.0 (13)	27.0 (9)	0.75	33.0 (15)	27.0 (8)	0.29
*Age groups, %:*						
18–20	45	31		35	31	
21–24	11	29	0.41	9	32	0.06
25–29	11	22		21	21	
30–34	33	18		35	16	
Current breastfeeding, %	33	21	0.40	26	21	0.59
Marital status – Divorced/separated, %	22	12	0.32	13	13	1.0
Marital status – Never married, %	56	66	0.72	61	66	0.64
Marital status – Widowed, %	22	22	1.0	26	21	0.59
Have HIV positive sex partner, %	11	8	0.53	9	4	0.69
Median no. years in sex work (IQR)	2.5 (6.5)	4.0 (3)	0.48	4 (5)	4 (3)	0.94
History of forced sex	44	37	0.73	39	37	0.87
Lifetime HIV testing history, %:						
Never tested	22	52		48	50	
Once	44	30	0.01	13	34	0.02
Twice	0	14		17	12	
3–5 times	22	5		17	3	
≥6 times	11	1		5	1	
Had HIV test in past 6 months, %	11	2	0.18	4	2	0.42
≥1 AIDS symptom in last 6 months+, %	33	43	0.73	22	19	0.77
Median baseline CD4 cells/µl (IQR)	447	461	0.77	590	444	<0.01
Classified as RI by baseline STARHS test during cross-sectional survey, %	100	0	<0.0001	65	17	<0.0001
Median no. days between baseline and repeat STARHS test	692	623	0.69	593	641	0.42

Abbreviations: RI = recent infection; LTI = long-term infection.

1 P-values for the Chi-square or Fisher's exact tests for categorical variables, and the Wilcoxon-Mann-Whitney test for continuous variables.

+ Includes: recent unexpected weight loss, chronic diarrhea, chronic weakness, fever, cough, night sweats, oral candidiasis.

### Unadjusted and adjusted STARHS incidence rate estimates

Unadjusted HIV incidence estimates based on the BED, Ax-AI, and combined BED/Ax-AI algorithm were: 6.5 infections per 100 person-years (PY) (95% CI: 3.2–9.9), 10.8 per 100 PY (95% CI: 5.6–16.0), and 5.2 per 100 PY (95% CI: 2.2–8.1), respectively (Table 2). Adjustment of assay-based incidence estimates with their corresponding FRR reduced the BED, Ax-AI, and combined BED/Ax-AI estimates to: 5.5/100 PY (95% CI: 2.2–8.7); 7.7/100 PY (95% CI: 3.2–12.3); and 4.4/100 PY (1.4–7.3), respectively. Exclusion of individuals with CD4 count <200 cells/µl from RI classification (without adjustment by FRR) reduced the BED, Ax-AI, and combined BED/Ax-AI estimates to: 5.6/100 PY (95% CI: 2.6–8.6); 9.7/100 PY (95% CI: 5.0–14.4); and 4.7/100 PY (95% CI: 2.0–7.5), respectively.

**Table 2:** HIV incidence estimates based on STARHS assays among ARV-naïve, high-risk women in Kigali, Rwanda.

Assay	Number HIV positive	Number Recent	Number HIV negative	Assay window period^1^	Estimated Incidence
**Unadjusted estimates**					
BED-CEIA	190	36	610	330	6.5 (3.2, 9.9)
AxSYM Avidity Index	190	56	610	310	10.8 (5.6, 16.0)
BED and Ax-AI	190	23	610	267	5.2 (2.2, 8.1)
**CD4-adjusted estimates, cut-off ≥200 cells/µl** 2					
BED-CEIA	190	31	610	330	5.6 (2.6, 8.6)
AxSYM Avidity Index	190	50	610	310	9.7 (5.0, 14.4)
BED and Ax-AI	190	21	610	267	4.7 (2.0, 7.5)
**Adjusted with local BED false-recent rate of 3.6%** 3					
BED-CEIA	190	36	610	330	5.5 (2.2, 8.7)
**Adjusted with local Ax-AI false-recent rate of 10.6%** 3					
AxSYM Avidity Index	190	56	610	310	7.7 (3.2, 12.3)
**Adjusted with local BED/Ax-AI combined false-recent rate of 2.1%** 3					
BED and Ax-AI	190	23	610	267	4.4 (1.4, 7.3)

1. Sample-specific window periods, based on data from seroconverter panel.

2. CD4 adjustment removes individuals with CD4<200 from recent infection classifications: 2 from Concordant; 5 from BED-RI.

3. False-recent rate calculations exclude individuals taking antiretroviral therapy and with CD4 count <200 cells/µl. Assuming a CoV for the window period of 20%; (CoV for FRR were calculated and input into spreadsheet).

### Comparison between STARHS incidence estimates and estimated cohort incidence rate

**Figure 3:**
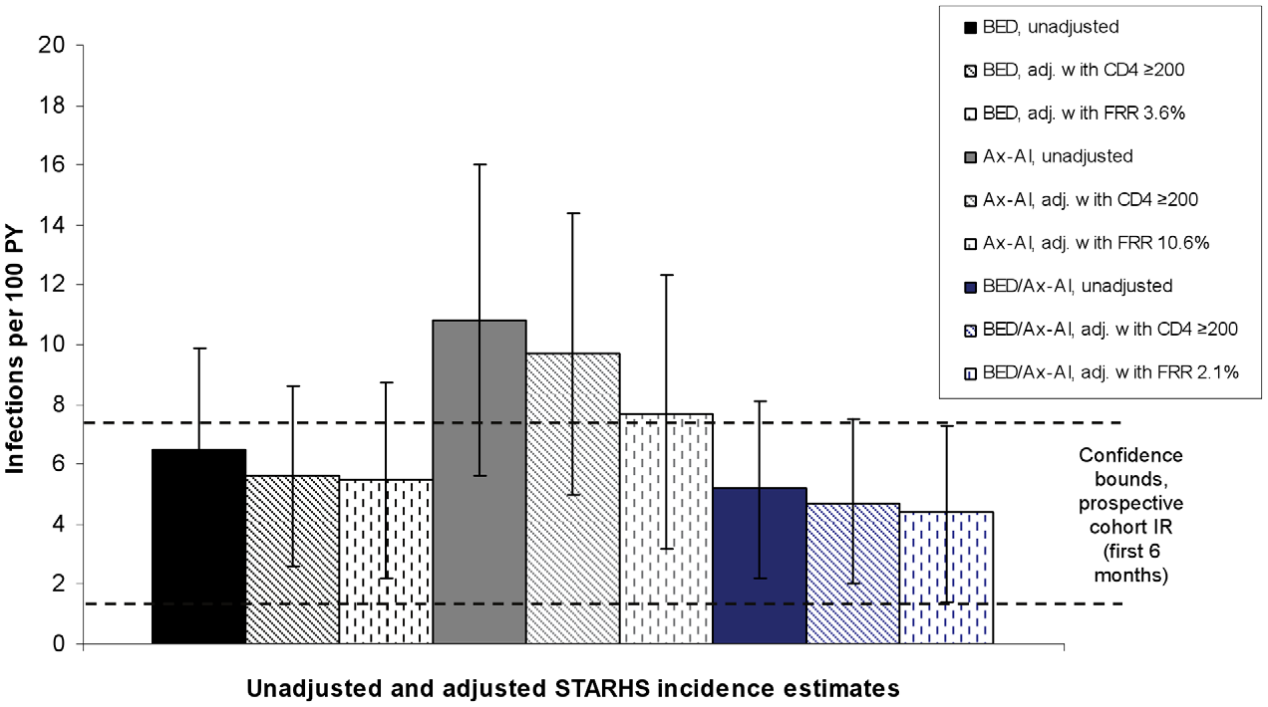
STARHS assay-based incidence estimates, cross-sectional sample.

In the prospective cohort sample, the estimated 12-month HIV incidence rate was 3.5 infections per 100 PY (95% CI: 1.6–5.4). However, HIV incidence showed a non-significant downward trend over time, with rates of 4.6/100 PY (95% CI: 1.6–7.7) and 2.2/100 PY (95% CI: 0.1–4.4) in the first and second 6 months of the cohort, respectively. Compared with the highest cohort incidence rate (from the first 6 months of the cohort), all STARHS-based incidence estimates for the cross-sectional sample fell within the confidence bounds of the estimated prospective cohort rate, except for the unadjusted Ax-AI estimate and the CD4-adjusted Ax-AI estimate (Fig. 3). The FRR-adjusted Ax-AI estimate fell nearly within the CI of the prospective rate.

## Discussion

In this investigation, incidence estimates based on cross-sectional data were affected by misclassification by the BED and Ax-AI assays. The unadjusted BED (6.5/100 PY) and Ax-AI (10.8/100 PY) incidence estimates were both substantially higher than our estimated prospective cohort 12-month incidence rate of 3.5/100 PY (95% CI: 1.6–5.4). Even after adjustment for poor specificity with CD4 data or sample-specific FRR, most assay-based estimates remained substantially higher than the overall estimated incidence rate for the prospective cohort. However, comparison with the incidence rate for the first 6 months of the cohort showed good correspondence with most STARHS-based estimates, particularly those for the combined BED/Ax-AI algorithm (both unadjusted and adjusted).

Comparisons between incidence estimates derived from the cross-sectional and prospective samples may be fraught for several reasons. First, by definition, cross-sectional and prospective incidence rates are estimated at different time points (over some time prior to baseline and during the months following baseline, respectively). Second, limited statistical power, as in the case of this analysis, will make meaningful comparisons between estimates difficult. Third, CD4- and FRR-adjustment strategies may not have fully corrected for misclassification in the cross-sectional sample, thereby leaving residual bias in the BED and Ax-AI incidence estimates. Fourth, selection bias may have caused the estimates to diverge, for example if there were differences in risk between women who enrolled in the prospective cohort and women in the survey sample who were eligible (i.e., HIV negative) but did not enroll. Indeed, cohort participants tended to be lower risk than non-enrolled women (data not shown). In addition, observation biases such as the Hawthorne effect or study-related risk-reduction interventions (e.g., condom provision, prevention counseling, STI treatment) may create artifactual differences in rates between prospective and cross-sectional samples[1]. We did observe a non-significant downward trend in incidence in the prospective cohort during follow-up, which could be due to the Hawthorne effect and/or some effect of study interventions. While specific reasons for the downward trend are difficult to isolate, the trend supports using early (first 6 months) rates from the cohort as the most appropriate comparator for STARHS-based estimates.

The BED-FRR in this sample was lower than BED-FRR reported for Zimbabwean[9] and North American[8] samples, but was higher than the rate reported for a rural South African sample[18]. Compositional, clinical (e.g., circulating HIV subtype or ART coverage), or biologic differences (e.g., disease progression) among the study populations could explain differences in the FRR. To our knowledge, this is the first publication of a false-recent rate for the Ax-AI method based on follow-up STARHS testing of HIV-positive survey participants. In this study, the FRR of the Ax-AI method was higher than the FRR for the BED assay. Although few studies have compared and contrasted results from the two assays, one study in Côte d'Ivoire did report poorer specificity on the Ax-AI as compared to the BED in prospective study seroconverter panels[7]. Poorer performance of the Ax-AI in this study, including the low correlation with BED, could be due to suboptimal cross-reactivity with a range of HIV-1 subtypes[24].

Estimated mean window periods for the BED and Ax-AI assays among participants in the prospective sample were substantially longer than published window period values (330 vs. 155 days for BED, and 310 vs. 180 days for Ax-AI). Differences in mean window period may reflect underlying variability in the biologic response after infection with different HIV-1 subtypes[10], [25]. Indeed, a recent analysis of data from multiple HIV seroconversion cohorts with varying HIV subtypes estimated the overall mean BED window period to be 197 days, with longer window periods for African vs. non-African cohorts (Parekh et al., *submitted*). While the small sample size and lack of robust methods led to a high degree of uncertainty in our sample-specific window period estimates, they suggest potential improvement in assay performance with longer window periods—a finding that underscores the benefit of using a locally derived window period. For example, using the manufacturer's window period of 155 days for BED, the unadjusted incidence estimate is 13.9/100 PY (95% CI: 6.7–21.0), versus 6.5/100 PY (3.2, 9.9) with our estimated local window period of 330 days. An ideal assay would be applicable to all HIV-1 subtypes, as well as not rely on modifying existing commercial assays, be easy to transfer in the field, and be unaffected by changes in HIV antigen-specific antibodies associated with long-term infection.

This is also the first report of a false-recent rate for the combined BED/Ax-AI algorithm. The FRR for the combined BED/Ax-AI algorithm (among ART-naïve participants) was lower than the individual assay FRR, at only 2.1%. Indeed sequential testing with two STARHS assays is increasingly being recommended as a strategy for reducing misclassification and improving incidence estimates[26], [27]. However, with two assays that perform sub-optimally in a given population there will be a trade-off between improved specificity and loss of sensitivity[8]. Availability of ART has rapidly increased in Rwanda during the past few years[28]. As individuals taking ART may be misclassified on STARHS assays because of changes in HIV antibody level due to treatment, misclassification rates in this population may increase over time as more individuals initiate treatment[18]. The ART status of survey participants, especially those testing recent on assays, should be measured systematically (e.g., therapeutic drug monitoring (TDM), chart review, self-report) so that individuals taking ART can be excluded from incidence analyses and FRR calculations[29].

Several factors were associated with testing false-recent on the assays among HIV-positive participants with known LTI, including having been classified as RI by the assays at baseline; more frequent history of HIV testing; and older age (borderline significant association). HIV testing history and older age were significantly positively associated with long-term HIV infection in this sample (data not shown). The association between testing false-recent and having a prior STARHS classification of RI may reflect the presence of “assay non-progressors” in this sample, or individuals who are repeatedly classified as RI by STARHS assays over time because of sustained low antibody levels[26]. Further, false-recent classification by Ax-AI could be due, in part, to infection with multiple HIV clades, wherein subsequent waves of antibody production maintain low antibody avidity[30]–[32]. Our observation of a higher Ax-AI-false recent rate among participants with an HIV-positive partner, frequent HIV testing history, and higher baseline CD4 count, support such a hypothesis.

In this population, adjustment of STARHS-based incidence estimates with FRR brought estimates closer to the gold standard estimated cohort incidence rate than did adjustment using a CD4 cutoff of <200 CD4 cells/µl for probable LTI. Incidence surveys should use a locally derived, population-specific FRR versus a published rate from a different population, and indeed should be reconsidered when a local FRR is not available. While follow-up of a long-term infection cohort such as was done in this study is the optimal method for estimating an FRR, false-recent rates can also be estimated in sufficiently large cross-sectional samples of ART-naïve individuals with long-term infection. In our study, CD4 adjustment also appeared to help reduce potential inflation of estimates, which underscores the value of CD4 data for adjusting and interpreting STARHS results, and thus the importance of incorporating CD4 count measurement into national or population-based serosurveys using STARHS to estimate HIV incidence if feasible. CD4 testing may be feasible, for example, in settings with enhanced clinical and laboratory capacity as a result of treatment scale-up. Ideally, assay FRR and CD4 count data, along with other clinical information, would be available for adjusting STARHS-based incidence estimates.

This study has several strengths. The combined cross-sectional and prospective design enabled us to compare incidence estimates, derive population-specific FRR on the assays, including for the combined test algorithm, and estimate assay window periods from serial specimens from individuals with known interval of HIV seroconversion. The use of two STARHS assays contributes important information about the assays' independent and relative performance in a high-risk setting with little experience with STARHS. Discussion is ongoing regarding the optimal assay parameters (e.g., window periods, cutoff values, including use of a “grey zone” instead of a single value) for a combined BED/Ax-AI algorithm.

Study limitations are also noted. The small sample size of the study, and relatively few HIV seroconversions and recent infection classifications, may have limited statistical power for certain analyses. Specifically, the small sample size, along with other study design features, prohibited the use of more robust statistical methods for comparing the cross-sectional and prospective incidence rates, such as equivalence tests[33], and may have also led to reduced precision around the assay FRR estimates[9]. However, the statistical approach employed for incidence estimation does attempt to quantify the affect of uncertainty in the calibrating parameters (e.g., FRR and window period) on the incidence estimates. Furthermore, our approach to CD4-adjustment of STARHS classifications (using a cutoff of <200µl for LTI) may have erroneously excluded individuals with primary HIV infection and low CD4 count[34] from the RI classification. However, the lower limit of 287 CD4 cells/µl among recent seroconverters in this sample suggests that using a cutoff of CD4<200 for adjustment would not result in the loss of many individuals with true RI status in incidence estimates (indeed there were no individuals with CD4<50 and RI status on the assays). Additionally, participants' ART status was assessed by self-report rather than by pharmacokinetic testing. However, women in the baseline survey were newly diagnosed with HIV by the study and so were assumed to be ART-naïve, and even at follow-up few women would have begun taking ART given the relatively short time since diagnosis. Finally, although some studies have shown that the Ax-AI method may be more specific than the BED assay[35], [36], the ideal dual testing algorithm would include a confirmatory test with perfect specificity.

In this sample of Rwandan FSW, adjusted incidence estimates based on a combined BED/Ax-AI algorithm were similar to the estimated HIV incidence rate in the first 6 months of cohort follow-up, when incidence was highest. Furthermore, false-recent rate on the combined BED/Ax-AI algorithm was low, and substantially lower than for either assay alone. In population-based testing, specificity of the BED and Ax-AI assays, and the combined test algorithm, would be expected to be substantially higher given that a larger proportion of individuals will have longer-term HIV infection.
